# Incorporation of Multiple-Days Information to Improve the Generalization of EEG-Based Emotion Recognition Over Time

**DOI:** 10.3389/fnhum.2018.00267

**Published:** 2018-06-29

**Authors:** Shuang Liu, Long Chen, Dongyue Guo, Xiaoya Liu, Yue Sheng, Yufeng Ke, Minpeng Xu, Xingwei An, Jiajia Yang, Dong Ming

**Affiliations:** ^1^Neural Engineering & Rehabilitation Lab, Department of Biomedical Engineering, College of Precision Instruments and Optoelectronics Engineering, Tianjin University, Tianjin, China; ^2^Academy of Medical Engineering and Translational Medicine, Tianjin University, Tianjin, China

**Keywords:** emotion, electroencephalogram (EEG), generalization, emotion recognition, day-to-day variations

## Abstract

Current studies have got a series of satisfying accuracies in EEG-based emotion classification, but most of the classifiers used in previous studies are totally time-limited. To produce generalizable results, the emotion classifier should be stable over days, in which the day-to-day variations of EEG should be appropriately handled. To improve the generalization of EEG-based emotion recognition over time by learning multiple-days information which embraces the day-to-day variations, in this paper, 17 subjects were recruited to view several video clips to experience different emotion states, and each subject was required to perform five sessions in 5 days distributed over 1 month. Support vector machine was built to perform a classification, in which the training samples may come from 1, 2, 3, or 4 days' sessions but have a same number, termed learning 1-days information (L1DI), learning 2-days information (L2DI), learning 3-days information (L3DI), and learning 4-days information (L4DI) conditions, respectively. The results revealed that the EEG variability could impair the performance of emotion classifier dramatically, and learning more days' information to construct a classifier could significantly improve the generalization of EEG-based emotion recognition over time. Mean accuracies were 62.78, 67.92, 70.75, and 72.50% at L1DI, L2DI, L3DI, and L4DI conditions, respectively. Features at L4DI condition were ranked by modified RFE, and features providing better contribution were applied to obtain the performances of all conditions, results showed that the performance of SVMs trained and tested with the feature subset were all improved for L1DI, L2DI (^*^p < 0.05), L3DI (^**^p < 0.01), and L4DI (^*^p < 0.05) conditions. It could be a substantial step forward in the development of emotion recognition from EEG signals because it may enable a classifier trained on one time to handle another.

## Introduction

Emotion is a psycho-physiological process triggered by the conscious and/or unconscious perception of an object or a situation, which is often associated with mood, temperament, personality disposition, and motivation (Koelstra et al., 2012). It plays a key role in non-verbal communication, and it is essential to understand human behavior. Emotion recognition has recently received an increasing amount of attention in human-computer interface and affective disorder diagnosis (Acharya et al., 2015; Atkinson and Campos, 2015; Yin et al., 2017). Through measuring the human signals, operators could recognize the current emotion state just by the automatic emotion recognition system. So it could also be used to many other applications such as driving safety, entertainment, e-learning, and telemedicine (Nasoz et al., 2004; Liu et al., 2010). Though emotion recognition has been traditionally done from facial expressions, speech or gesture, these signals have a critical limitation in that these could be deliberately changed to hide the true emotion (Yoon and Chung, 2013). Physiological measures, such as electroencephalogram (EEG), electrocardiogram (ECG), electromyogram signal, respiratory volume, and skin conductance, have been widely used recently because of its objective (Khalili and Moradi, 2008; Kim and André, 2008; Koelstra et al., 2012; Yoon and Chung, 2013). Among these, non-invasively measured EEG has been a growing popular tool with the advantages of high time resolution as well as simple and affordable recording requirement (Xu et al., 2016).

A variety of EEG features have been employed in emotion recognition so far. EEG power spectrum distributions has been repeatedly reported to be a discriminable marker of emotions. Specifically, frontal asymmetry in the alpha band has been used as a predictor of valence (Lee et al., 2014). More recently, nonlinear features have been proposed to recognize emotions, such as fractal dimensions (Sourina et al., 2009; Liu et al., 2010; Ahmadlou et al., 2012), Hurst exponent (Wang et al., 2014; Acharya et al., 2015) and entropy (Duan et al., 2013; Wang et al., 2014), wavelet-chaos methodology (Ahmadlou et al., 2012). Furthermore, phase synchronization and coherence (Miskovic and Schmidt, 2010; Martini et al., 2012) have also been considered as emotional features.Regarding the classification, there have been lots of machine learning methods producing excellent performance, such as the support vector machine (SVM) (Brown et al., 2011; Nie et al., 2011; Soleymani et al., 2012; Duan et al., 2013; Hidalgo-Muñoz A. et al., 2013; Hidalgo-Muñoz A. R. et al., 2013), k-nearest neighbor (Guyon and Elisseeff, 2003) multilayer perceptron (Yoon and Chung, 2013), linear discriminant analysis (Murugappan et al., 2010) and Bayesian network (Hidalgo-Muñoz A. et al., 2013; Hidalgo-Muñoz A. R. et al., 2013), and so on. Current studies got a series of satisfying accuracies in emotion classification from EEG signals, but most of the classifiers are time-limited. It is however impossible that an emotion classifier trained on the data at specific time can only recognize emotion state at the same time in practical application.

As we know, a person' EEG patterns may appear differently at different time even when he is under the same emotion due to some external factors such as temperature, humidity, or a diet, and also some uncontrollable internal factors such as the hormones or baseline mood that can cause variations in physiology (Chueh et al., 2012). The reliability of resting EEGs over time has already been studied for decades. The correlation coefficients were reported to decrease with the test-retest interval increasing, and power spectral parameters were proved to be more stable than others such as entropy and coherence features (Gasser et al., 1985; Salinsky et al., 1991; Kondacs and Szabó, 1999; Gudmundsson et al., 2007). It seems that the stability of emotional EEG features has not been substantially addressed until very recently. Liu et al. (Lan et al., 2014) used the Intra-class Correlation Coefficient (ICC) to quantified the stability of four feature parameters regarding emotion recognition, respectively, but it did not present a way to solve the day-to-day variations of EEG. An emotion classifier does not generalize over days if the day-to-day variations is not appropriately handled. If a classifier is built by the data drawn from just 1 day, the input features may carry the information unique to that day which the classifier would learn. Once the testing set was independently from another day, the performance of the classifier was undermined. Up to now, researchers have not yet reported the effort to handle this issue in EEG-based emotion recognition.

This study aims to investigate the influence of EEG's day-to-day variations on the performance of an emotion classifier, and the benefit of the multiple-days information to constructing a classifier generalizing over time. This paper is organized as follows. Section Materials and Methods addresses the methodology including the experiment and data processing. Section Results shows the results. The discussion and conclusion are stated in section Discussion and Conclusion.

## Materials and methods

### Materials

#### Participants

The experiment was performed with 17 healthy participants (12 female, 5 male, age range 20–28). All participants had normal or corrected-to-normal vision and normal hearing, and none of them had a history of severe medical treatment, psychological or neurological disorders. A signed consent was obtained from each subject before the experiment was carried out.

#### Stimuli and experimental procedure

A group of emotional movie clips were used to evoke subject's neutral, positive, and negative states. To evaluate the effectiveness of these clips, 30 subjects took part in a questionnaire survey and the final 45 movie clips with strongest ratings and a small variation were selected for use in the experiment. The spoken language of the clips is Chinese or dubbed into Chinese with the length of from 5 to 20 min.

**Figure 1:**
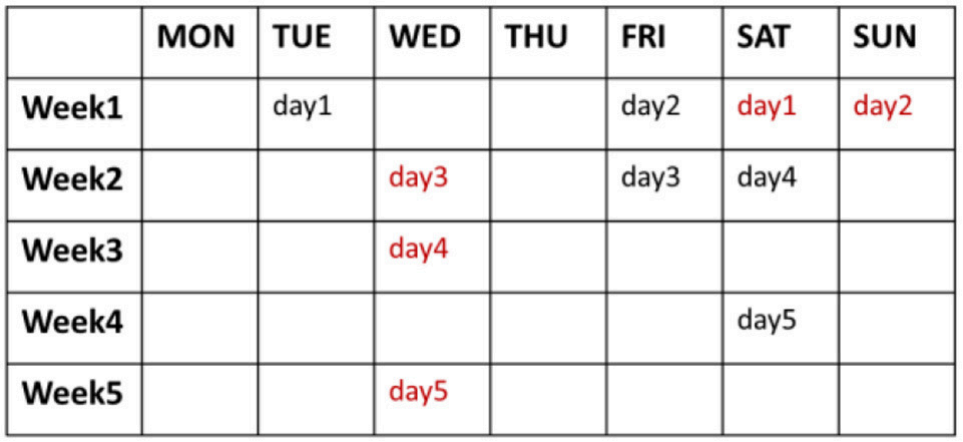
Data collection days for two representative subjects.

Before the experiment, each subject was informed of the experimental procedure and the meaning of valence and arousal used for self-assessment. The subjects were required to perform five sessions in 5 days distributed over 1 month, with 6–9 video clips in each recording session. Recording sessions for two representative subjects are depicted in Figure 1. The number of days between consecutive sessions was in a random order of four intervals: 1 day apart, 3 days apart, 1 week apart and 2 weeks apart intervals. This randomization was intended to reduce the effect of strategic changes. The participants were invited to the listening room between 18:00 p.m. and 20:00 p.m. and presented 6–9 clips for every recording session, ensuring there were 2 clips successfully eliciting positive, neutral and negative emotion states, respectively. After watching a video, the participants were required to score their feelings on a 9-point scale in terms of emotional valence and arousal, and then had a short break. Subjects were informed to report what they actually felt during watching movie clips, not what they thought they should feel.

#### EEG recording and preprocessing

**Figure 2:**
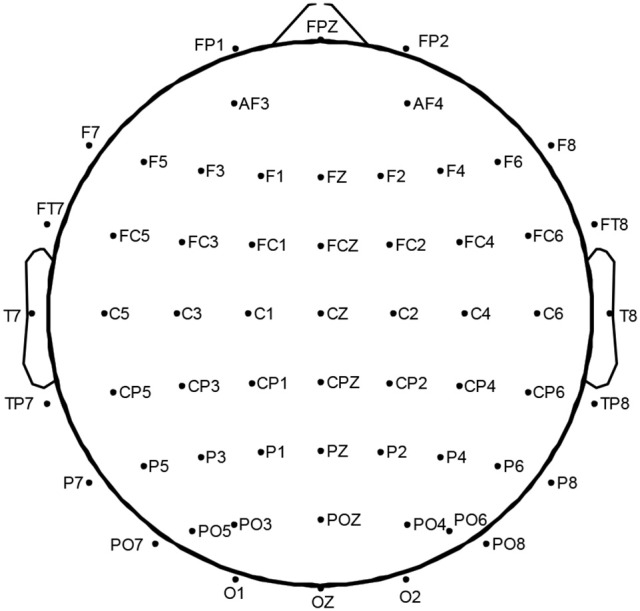
International 10–20 system for 60 electrodes.

During watching the clips, EEG data were recorded continuously using 60 sites using a NeuroScan SynAmps^2TM^, positioned following the international 10–20 system (Tytell, 1961). Figure 2 shows 60-channels EEG cap layout used in this study. Right mastoid was used as reference, and the central region was used as the grounding site. The EEGs were digitized at 1,000 Hz and filtered at 0.1–100 Hz.

A preprocessing step was performed for the raw EEG signals. All channels were re-referenced to bilateral mastoid, and down-sampled to 500 Hz. EOG artifacts were removed using independent component analysis (ICA) (Li et al., 2006). Data were segmented according to the subjects' self-report about which period of time they felt a strong emotional experience.

### Methods

#### Feature extraction

EEG power spectrum distributions in various frequency bands have been repeatedly reported to be a discriminable marker of emotions (Balconi and Lucchiari, 2006; Lee et al., 2014; Verma and Tiwary, 2014). In this paper, all of the 60-channel data were spectrally analyzed using Welch's method with a hamming widow of 500 ms with 50% overlap. Then the spectral powers in delta band (0.5–4 Hz), theta band (4–8 Hz), alpha band (8–13 Hz), beta band (13–30 Hz), low gamma band (30–44 Hz), and high gamma band (44–100 Hz), were computed, resulting in 360 total features (6 per channel × 60 channels), and 5-s epoch was extracted as a sample. The number of extracted samples depended on the feedback data of the subject, but equal numbers of samples were used to train the classifier, this would be described in detail below.

#### Different strategies of classifier calibration

This work tried to investigate the influence of EEG's day-to-day variations on the performance of an emotion classifier firstly. To this end, two different methods, within-day classification (WDC), the standard cross-day classification (SCDC) were compared.

(1) WDC: The training and the testing data were all from the same day. Eighty percent of the data samples from test day were randomly selected and used for classifier training, while the remaining 20% were sent to the testing set. To obtain a robust result, this procedure was repeated 5 times, and the classification rates were got by averaging all the 5 accuracies. Each day was treated as the test day once.
(2) SCDC: The training and testing data were from two independent days. Eighty percent of the data samples from 1 day were randomly selected as the training data, and the data from remaining 4 days were sent to test the classifier, respectively. Each day was used to train a classifier once. The number of training samples in both WDC and SCDC was equal to ensure fair and valid comparisons.

#### Learning multiple-days information for classifier calibration

**Figure 3:**
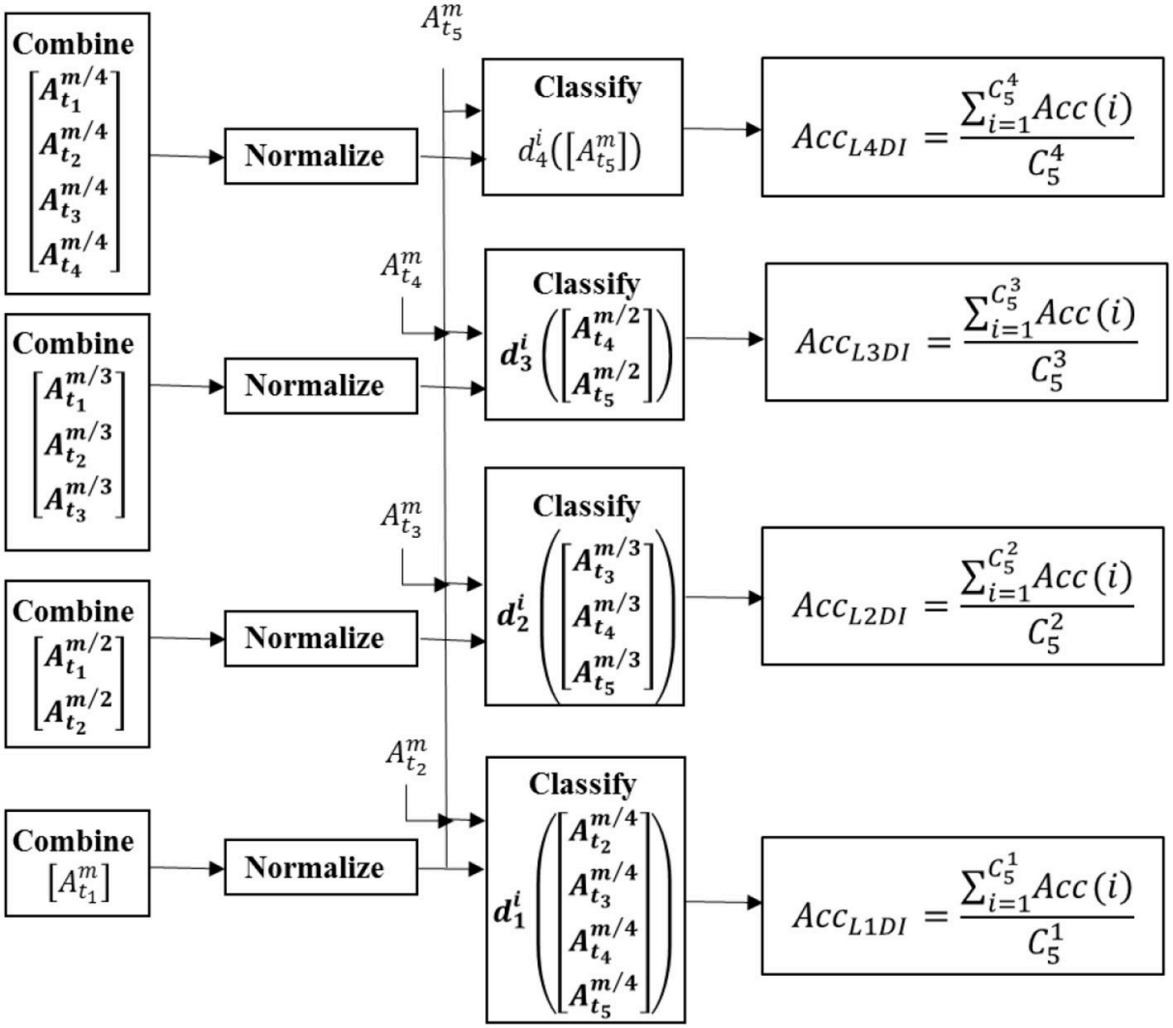
The flow charts of LNDI.

In order to verify the benefit of the multiple-days information to constructing a classifier generalizing over time, the classifiers were built by learning N-days information (*N* = 1, 2, 3, 4) and compared, termed as L1DI, L2DI, L3DI, and L4DI, respectively. For LNDI, the classifier learned N-days information, and data samples from the remaining 5-N days were sent to the testing set. Figure 3 shows the flow charts of LNDI.

Take L3DI as an example, the procedure was as follows:

The feature vector of the i*th* day was obtained by Welch's method (see section Feature Extraction) and represented by vector of $Y_{M_{i}}^{i}$ in form,

(1) $$\begin{array}{r} Y_{M_{i}}^{i}=\left[y\left(1\right);y\left(2\right);\ldots ;y\left(M_{i}\right)\right] \end{array}$$

Where *M*_*i*_ denotes the number of samples extracted from the ith day. The superscript refers the ith day and the subscript is the number of the vector.

(2) $$\begin{array}{r} M_{min}=floor\left(\frac{\min\left(M_{1},M_{2},M_{3},M_{4},M_{5}\right)}{12}\right)*12 \end{array}$$

Where *M*_*min*_ is the minimum number of data samples among these 5 days. Then 3 of 5 days to train a classifier and all possible combinations of training and testing days were taken into consideration to get a robust result and reduce the impact of any 1 day being an outlier. For the k*th* possible combination, the training set and testing set were constructed as follows:

(3) $$\begin{array}{r} Y^{k}=\left[Y_{M_{min}/3}^{t_{1}};Y_{M_{min}/3}^{t_{2}};Y_{M_{min}/3}^{t_{3}}\right] \end{array}$$

(4) $$\begin{array}{r} T^{k}=\left[Y_{M_{min}/2}^{t_{4}};Y_{M_{min}/2}^{t_{5}}\right] \end{array}$$

Where *Y*^*k*^ is the training set and *T*^*k*^ is the testing set for the k*th* possible combination, *t*_1_, *t*_2_, and *t*_3_ refer to the selected number of days to train a classifier, and *t*_4_, *t*_5_ were the number of days to test. The number of data samples were set to *M*_*min*_ both in training and testing set, and the same for L1DI, L2DI, L3DI, and L4DI.

(5) $$\begin{array}{r} \text{Acc}\left(\text{k}\right)=\text{SVM}\left(Y^{k},T^{k}\right) \end{array}$$

The accuracy of the k*th* possible combination was obtained by SVM (Chang and Lin, 2006).

The accuracy of L3DI was obtained by averaging all possible combinations,

(6) $$\begin{array}{r} Acc_{L3DI}=\frac{\sum_{k=1}^{C_{5}^{3}}Acc\left(k\right)}{C_{5}^{3}} \end{array}$$

To increase the reliability of the accuracy results, ten repetitions of abovementioned procedure were performed, and these ten classification rates were averaged to get the accuracy.

#### The specifically-designed recursive feature elimination (RFE)

**Figure 4:**
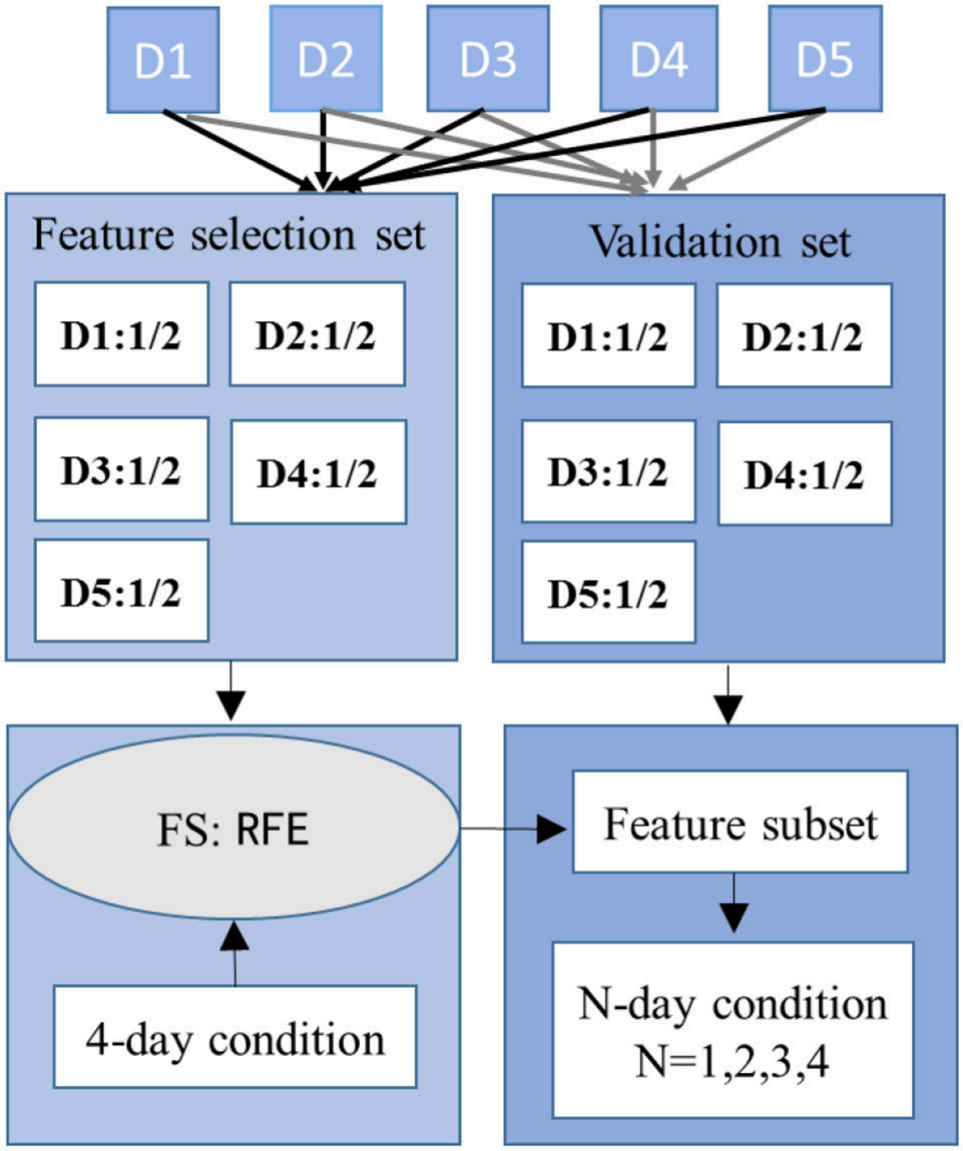
The illustration depicts a fold of the feature selection procedure.

To evaluate if learning multiple-days information (LMDI) could make the classifier easier to pick out the emotion-related features and discard day-specific ones, a specifically designed RFE was used for feature selection under L4DI condition. The method could rank all the features and then obtain the robust subset. The data samples were provided to the SVMs, split into training, testing, and validation sets. Fifty percent of the data samples from each day were randomly selected for feature selection, and the remaining 50% were held back as a validation set to control overfitting, as shown in Figure 4. The procedure can be summarized as follows:

(1) Fifty percent of the data samples from each day were randomly selected to construct the feature selection set, based on which 4 of 5 days were sent to train a classifier, the remaining 1 day were testing set. The number of training and testing samples followed the section Learning Multiple-Days Information for Classifier Calibration.
(2) To get the contribution ranking of features, firstly, one feature in N-dimension feature set was removed and then we computed the performances with remaining N-1 features; secondly, we removed each other feature once and then computed the performance with remaining N-1 features; Thirdly, we ranked the N performances got from the above two steps, and the feature owns the least contribution in the case that the corresponding performance has the minimum loss of accuracy.
(3) The top M of the feature ranking were considered as the robust features since these M features could achieve a relatively stable and high accuracy. As there were 5 combinations, so there were 5 feature lists with M features. Those selected 5, 4, and 3 times were considered features with better contribution and those with 2, 1, and 0 times were termed features with bad contribution. Salient feature subset was constructed by these features providing better contribution.
(4) The validation sets with the feature subset were then taken as input of the SVM to validate the feature subset under L1DI, L2DI, L3DI, and L4DI conditions, respectively.

## Results

### The EEG inter-day variability on emotion classification

**Figure 5:**
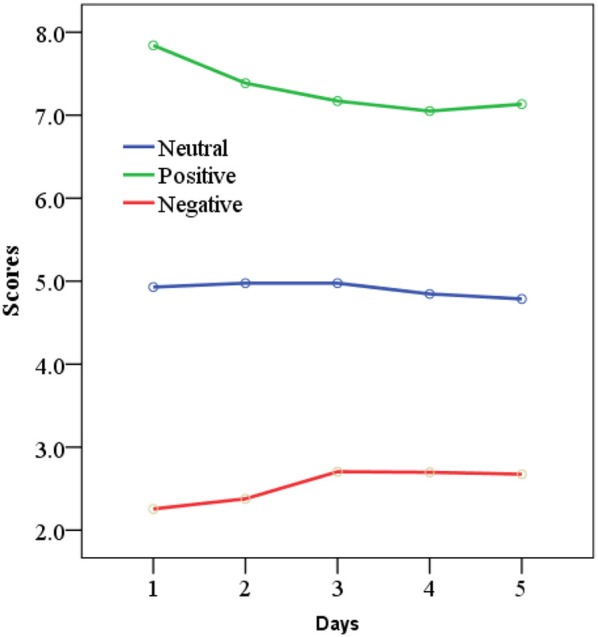
Valance ratings of neutral, positive, and negative states in 5-day experiment.

All the participants completed the 5-day experiment. All the rating scores (valence and arousal ratings) were collected, but we just concerned the valence dimension in subsequent analysis. Valance ratings of neutral, positive, and negative states were presented in Figure 5. To check our valence manipulation, 3 emotions by 5 days repeated-measure ANOVAs for valence ratings were performed. A main effect for three-class emotions on valence ratings was found, *F* = 1353.5, *p* < 0.001. The main effect for time (5 days) on valence ratings had no significance, *F* = 0.524, *p* = 0.718. This illustrated that the video clips have evoked the target emotions successfully, and there was no significant difference in valence ratings of 5 days.

### The EEG inter-day variability on emotion classification

**Figure 6:**
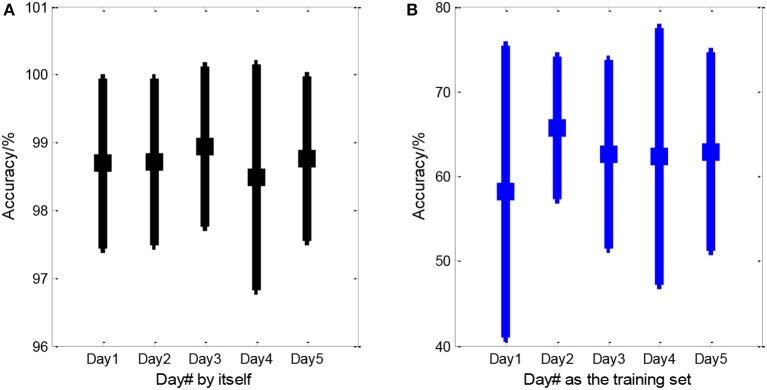
Averaged accuracies of within- and standard cross-day emotion classification. **(A)** Within-day classification and **(B)** the standard cross-day scenario that accounted for inter-day EEG variability. The spot indicates the mean value and the line is the standard deviation. In **(A)**, Day# presented the day that training samples and testing samples were both from. In **(B)**, Day# indicated the training day, and remaining 4 days were hold as the testing days.

We computed three-class (positive, neutral, and negative) accuracies of WDC and SCDC, shown in Figure 6. WDC is labeled when trained and tested on the same day, while SCDC is labeled when trained and tested on the different days, which is detailed in section Different Strategies of Classifier Calibration. As shown in Figure 6A, WDC returned the averaged accuracy of above 98%, which was impressive. Figure 6B shows the SCDC results using SVM, collapsed to about 60%, which presumably suffered from day-to-day variability. All classifiers are also well above chance performance.

**Figure 7:**
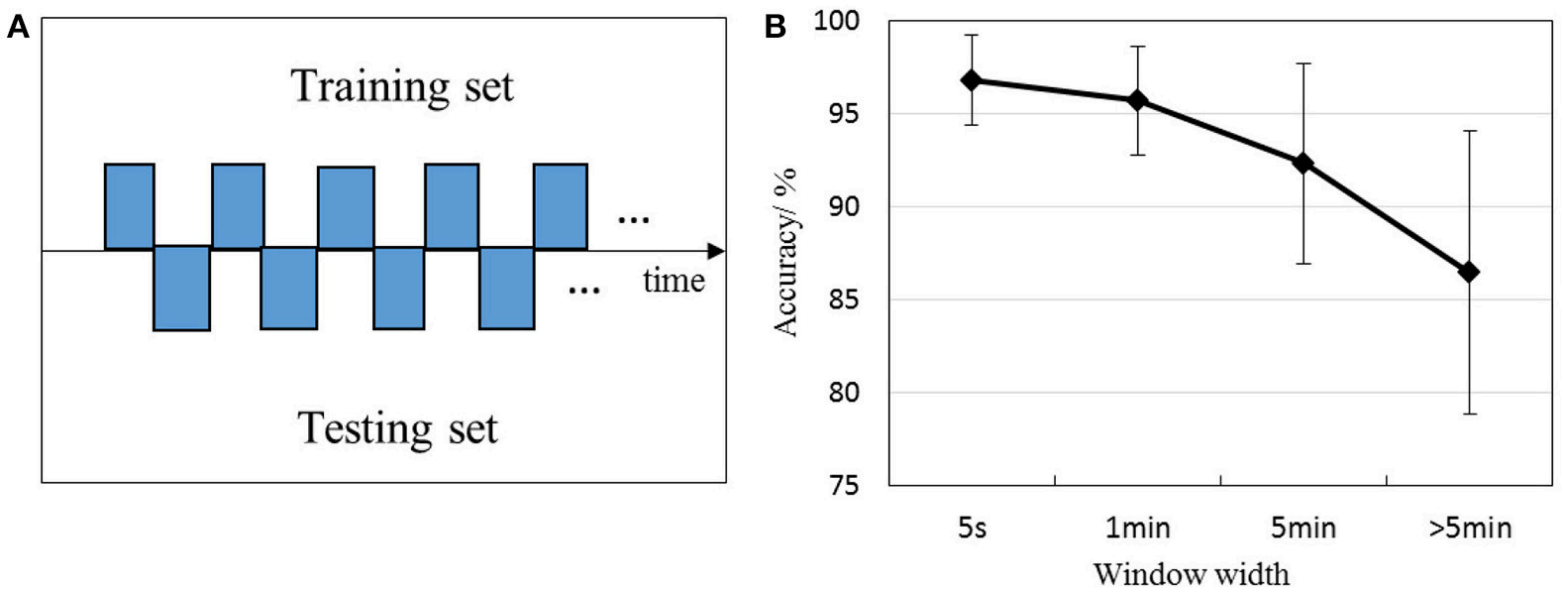
Classification rates with different window width. For example, 1 min represents the first 12 samples were sent to the training set, and the next 12 samples to the testing set in sequence, then the third 12 samples were to the training set. **(A)** Illustrates the sample partitioning method with a certain particular window width, **(B)** depicts the performance with different window width.

We calculated accuracies with different window width to further dissect the effect of EEG's day-to-day variations on the performance of emotion classification, shown in Figure 7. The data was split into several parts with a certain particular window width in each day, one part was sent to the training set, and the next part was to testing set in sequence, as depicted in Figure 7A. Window width were set to 5 s, 1 min, 5 min, and more than 5 min. More than 5 min was the condition that data at a certain emotional state in each day was split into two halves, one half was to the training set and the other half was to the testing set. Window width was from 5 to 20 min in this condition. As depicted in Figure 7B, the performance tends to decrease with larger window width. Paired *T-*test was performed on the accuracies between each two window width, we verified that all accuracies differ significantly (*p* < 0.05). These results underpinned that the EEG variability can deteriorate the performance of emotion classifier dramatically.

### Classification accuracies with different number of days in the training set

**Figure 8:**
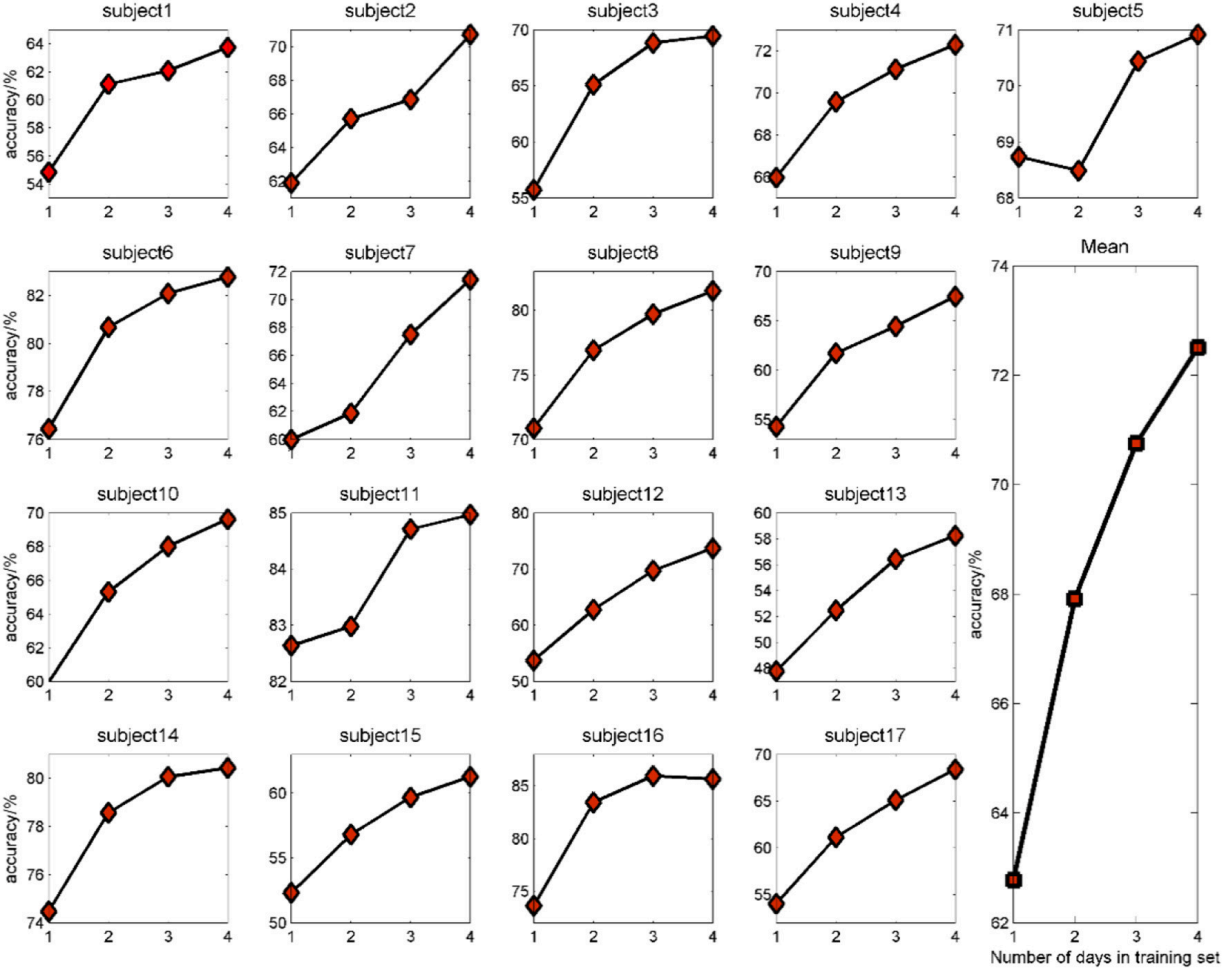
Classification accuracy with different number of days in the training set for all the subjects.

So we computed the accuracies of 4 conditions: L1DI, L2DI, L3DI, and L4DI conditions. Figure 8 shows the performance in 17 subjects, and the bottom right panel shows the mean accuracies across all subjects. As can be seen from Figure 8, the accuracies tended to increase with more days in the training set for all the subjects, the mean accuracies were 62.78, 67.92, 70.75, and 72.50% at L1DI, L2DI, L3DI, and L4DI conditions, respectively. Paired T- test revealed that the accuracy at L1DI condition was significantly lower than that of L2DI (*p* < 0.01), L3DI (*p* < 0.01), and L4DI conditions (*p* < 0.01). This confirmed the prediction that learning multiple-days information would improve the cross-day accuracies was correct.

### Performance with the feature subset applied to other conditions

Using data from more days to retrain a classifier could improve the accuracies over days, partly because it embraces the day-to-day changes incrementally. Thus the classifier trained by more days might weight emotion-related features heavily and inversely weaken the day-dependent features. The feature rank was obtained by modified RFE which detailed in the above.

**Figure 9:**
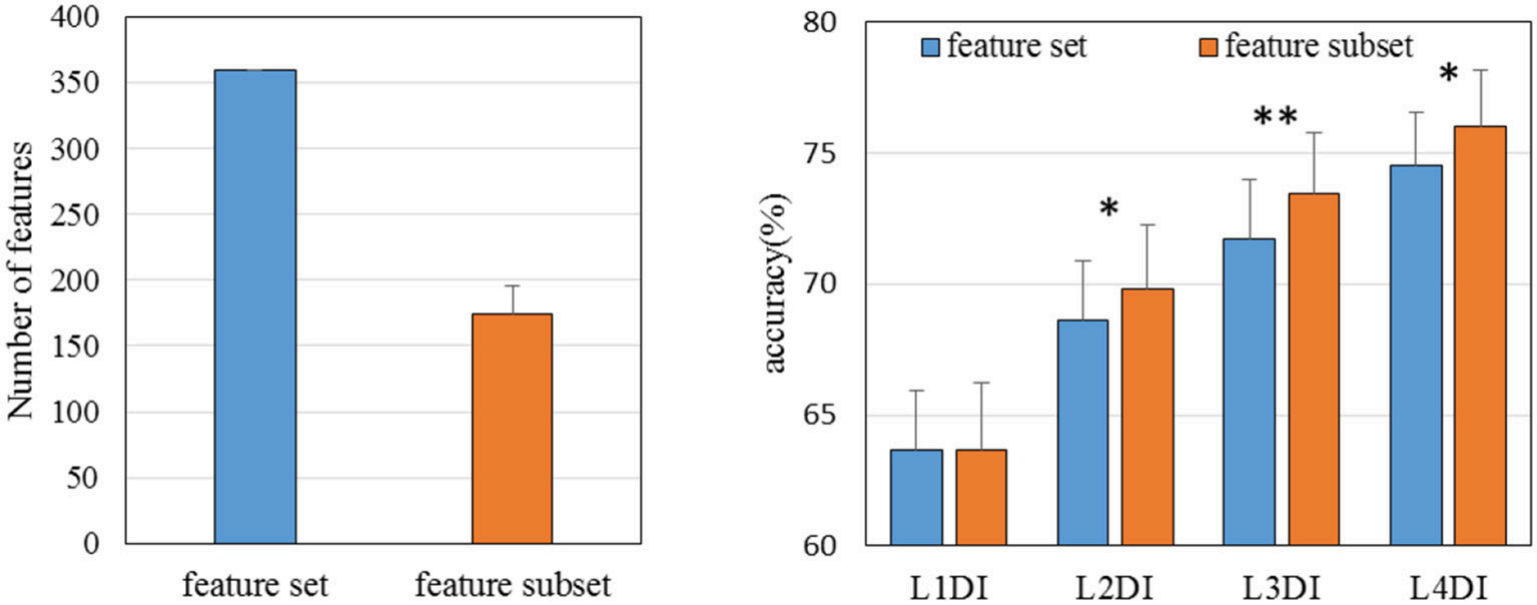
The performances of SVMs trained and tested with all 360 features and the feature subset (mean feature numbers was 174 across 17 subjects), respectively. *P < 0.05; **P < 0.01.

To check whether these feature subsets picked out by RFE at L4DI condition are emotion-related, and thus could benefit the performances of all conditions (L1DI, L2DI, L3DI, and L4DI conditions), the performances were computed on validation sets with all 360 features and the feature subset (mean number of the features was 174 across 17 subjects), respectively. As shown in Figure 9, compared with the performance of cross-day SVMs trained and tested with all the 360 features, the performance of SVMs trained and tested with the feature subset were all improved for L1DI, L2DI (^*^p < 0.05), L3DI (^**^p < 0.01), and L4DI (^*^p < 0.05) conditions, confirming the benefit of adding more days in training set for a classifier to weight emotional features heavily.

**Figure 10:**
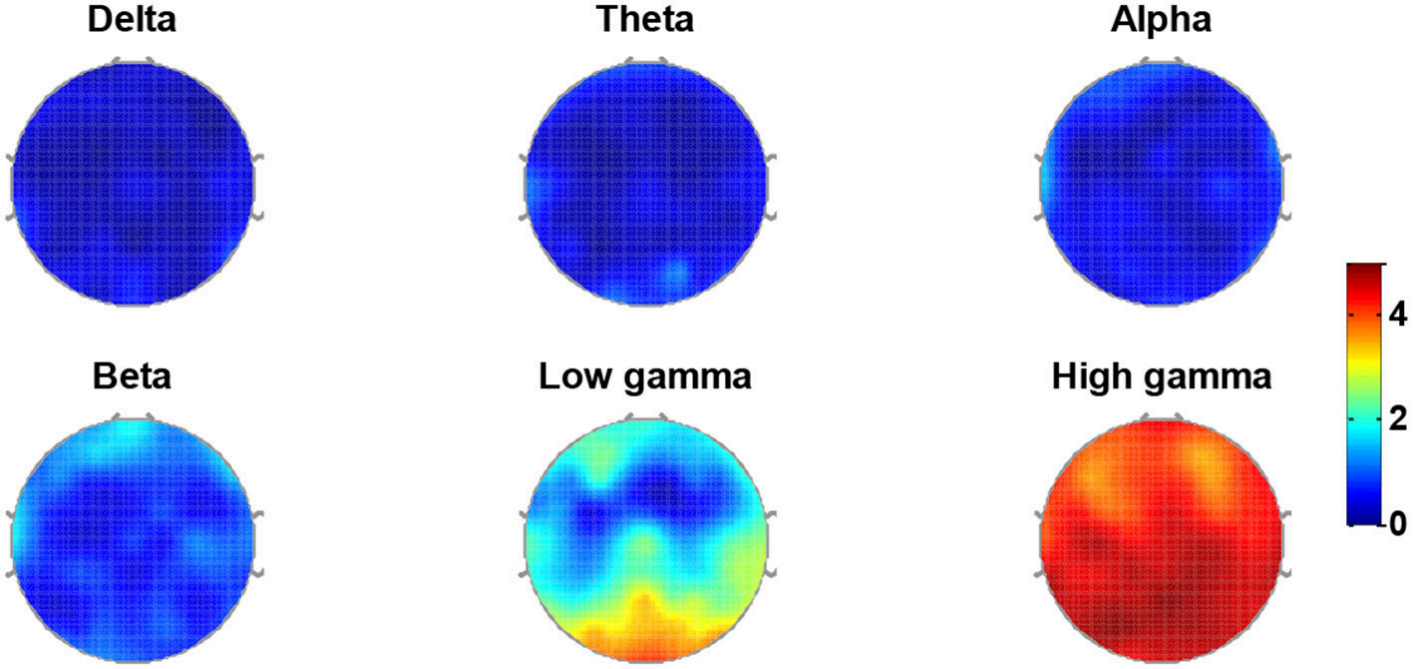
Topographic mapping of the quantified feature contributions averaged across 17 subjects for delta, theta, alpha, beta, low gamma, and high gamma bands.

Another issue that should be discussed is about the most emotion-relevant EEG frequency ranges. Figure 10 presents the distribution of the salient features averaged over 17 subjects. Contribution rate (CR) was computed for 64 channels in delta, theta, alpha, beta, low gamma, and high gamma bands, and depicted in Figure 10. It is obvious that the gamma band dominates a great proportion of the salient features, indicating gamma ranges might be importantly discriminable ranges for recognize the emotions.

### The sensitivity of the positive, negative and neutral valence

The confusion matrices in Table 1 provided a closer look at the sensitivity of the positive, negative and neutral valence. The row is the predicted label and the column is the real label. It could be found that positive, neutral and negative emotions were accurately recognized as 71.5, 76.9, and 59%, respectively. Moreover, there were a relatively higher proportion for negative valence that falsely classified as the neutral valence.

**Table 1:** Confusion Matrices under L4DI condition.

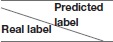	**Positive**	**Neutral**	**Negative**
Positive	0.715	0.144	0.1141
Neutral	0.077	0.769	0.154
Negative	0.128	0.282	0.590

## Discussion and conclusion

Results of this study demonstrated that classifiers that are trained and tested on EEG data from the same day can very accurately determine which emotion state produced the data. However the performance would be undermined by the day-dependent features when the testing samples were completely from an independent day, which was ignored by the existing studies. Prior work handling the day-dependent effect mainly focused on the feature selection. They often tried to find some robust feature which obtained better classification rates from a mass of features, but it did not solve the issue of the day-dependent effect thoroughly because the testing data took part in the procedure of feature selection. This study worked on emotion classification improvement by adding the day-to-day variability information into the emotion classifier. A novel classifier strategy, LMDI, was developed to include emotional EEG patterns from different days, which could improve the generalization of a classifier over time.

To avoid overfitting, the data were divided into two equal parts, one part for feature selection and the other for validation. Feature ranking was obtained under L4DI by RFE, and the top 100 features were considered to construct the robust feature subset. It has fortunately been found that accuracies could be significantly improved for all conditions (L1DI, L2DI, L3DI, and L4DI) with the relative low-dimension robust feature subset. These results showed that LMDI indeed improved the generalization of a classifier over time. It could be a substantial step forward in the development of emotion recognition from EEG signals because it may enable a classifier trained on one time to handle another.

All possible combinations of training and testing days were taken into consideration to avoid the impact of any 1 day being an outlier. An issue that should be discussed is whether the day-to-day variation of 1 day apart is smaller than more days apart. Take Day3 as the testing set for example, we obtained average accuracies of 60.8, 67.8, 61.4, and 68.5%, respectively for Day1, Day2, Day4, and Day5 as the training set. This implied the day-to-day variation is not gradually varied with the time, because the variation was caused by the change of EEG baseline, as well as the external condition such as electrode placements. The influence of day combinations on the recognition accuracies was also discussed here. Take Day 3 as the testing set for example as before, the results showed that there was no significant difference among the classification rates of different combinations respectively for L2DI (*p* > 0.1) and L3DI (*p* > 0.1).

In addition to their importance in emotion studies, day-dependent effect studies may also be indispensable in other fields such as mental workload, and other BCI systems. Therefore, more attention must be paid to day-dependence studies at both basic and applied levels. There are still challenges for future studies. A more generalized study protocol capable of handling different tasks, subjects and days should be a must.

## Ethics statement

This study was carried out in accordance with the recommendations of the ethical committee of Tianjin University. The protocol was approved by the ethical committee of Tianjin University. All subjects gave written informed consent in accordance with the Declaration of Helsinki.

## Author contributions

SL: conceptualization, planning, data collection, data analysis, and writing the manuscript. LC: conceptualization, data collection, and proofreading. DG: data collection and supporting data analysis. XL: data collection and proofreading. YS: supporting data collection. YK: conceptualization and supporting data analysis. MX: conceptualization, supporting data analysis. XA: supporting data analysis and proofreading. JY: proofreading. DM: conceptualization, planning, supporting data analysis, and proofreading.

### Conflict of interest statement

The authors declare that the research was conducted in the absence of any commercial or financial relationships that could be construed as a potential conflict of interest.
